# Cranial Nerve Development Requires Co-Ordinated Shh and Canonical Wnt Signaling

**DOI:** 10.1371/journal.pone.0120821

**Published:** 2015-03-23

**Authors:** Hiroshi Kurosaka, Paul A. Trainor, Margot Leroux-Berger, Angelo Iulianella

**Affiliations:** 1 Stowers Institute for Medical Research, Kansas City, MO, United States of America; 2 Department of Orthodontics and Dentofacial Orthopedics, Graduate School of Dentistry, Osaka University, Osaka, Japan; 3 Department of Anatomy and Cell Biology, University of Kansas Medical Center, Kansas City, KS, United States of America; 4 University Pierre and Marie Curie—Paris 6, Paris, France; 5 Department of Medical Neuroscience, Faculty of Medicine, Dalhousie University, Halifax, Nova Scotia, Canada; University of Colorado, Boulder, UNITED STATES

## Abstract

Cranial nerves govern sensory and motor information exchange between the brain and tissues of the head and neck. The cranial nerves are derived from two specialized populations of cells, cranial neural crest cells and ectodermal placode cells. Defects in either cell type can result in cranial nerve developmental defects. Although several signaling pathways are known to regulate cranial nerve formation our understanding of how intercellular signaling between neural crest cells and placode cells is coordinated during cranial ganglia morphogenesis is poorly understood. *Sonic Hedgehog* (*Shh*) signaling is one key pathway that regulates multiple aspects of craniofacial development, but whether it co-ordinates cranial neural crest cell and placodal cell interactions during cranial ganglia formation remains unclear. In this study we examined a new *Patched1* (*Ptch1*) loss-of-function mouse mutant and characterized the role of *Ptch1* in regulating *Shh* signaling during cranial ganglia development. *Ptch1^Wig/ Wig^* mutants exhibit elevated *Shh* signaling in concert with disorganization of the trigeminal and facial nerves. Importantly, we discovered that enhanced *Shh* signaling suppressed canonical *Wnt* signaling in the cranial nerve region. This critically affected the survival and migration of cranial neural crest cells and the development of placodal cells as well as the integration between neural crest and placodes. Collectively, our findings highlight a novel and critical role for *Shh* signaling in cranial nerve development *via* the cross regulation of canonical *Wnt* signaling.

## Introduction

The cranial nerves are part of the peripheral nervous system that governs various critical functions such as sensing and controlling movement within the craniofacial region. Previous studies in avian embryos have shown that some of the cranial nerves including the trigeminal (V) and facial nerves (VII) originate from both cranial neural crest cells and ectodermal placode cells [1,2]. Cranial neural crest cells arise in the dorsal neuroepithelium, delaminate via an epithelial to mesenchymal transformation, and migrate sub-ectodermally throughout the head and neck. In the peripheral nervous system, cranial neural crest cells generate neurons and glia. In contrast, ectodermal placodes comprise thickened regions of surface ectoderm cells, which are distinct from the neuroepithlium. Ectodermal placode cells delaminate from the surface ectoderm to establish the neurogenic core of the cranial nerves [3]. Cellular interactions between neural crest cells and placode cells are essential for proper cranial nerve patterning [4–6], and many signaling pathways influence cranial nerve formation in vertebrates by regulating cranial neural crest and/or ectodermal placode cell development [7]. However, our knowledge of how, and in what cell type or tissue these signals primarily function, and also how these different signaling pathways interact remains limited. This is due in part to the early embryonic lethality of many mutants in key developmental pathways.

In a previous study, we performed an N-ethyl-N-nitrosourea (ENU) mutagenesis screen in mice and identified multiple recessive alleles important for craniofacial development [8]. Here we characterize one of these ENU induced mutants called *Wiggable* (*Wig*) that carries a mutation in the *Patched1* (*Ptch1*) gene. *Ptch1* encodes a receptor for the Hedgehog family of morphogens which includes Sonic Hedgehog (Shh). Unlike *Ptch1* null mutant mice which are lethal at E9.5 [9], *Ptch1* ^*Wig/ Wig*^ mutants survive until E12.0, allowing an analysis of the effects of aberrant Shh signaling on cranial ganglia morphogenesis. In this study, we took advantage of multiple mouse mutants to clarify the role of cross-talk between the Shh and WNT signaling pathways during the formation of the trigeminal and facial nerves. We discovered that elevated *Shh* signaling restricts canonical *Wnt* signaling during cranial ganglia development. This affects the survival of migrating neural crest cells, the pattern of placode development and the integration between neural crest cells and placode cells. Our findings describe the importance of cross-talk between *Shh* and *Wnt* signaling in regulating tissue interactions during cranial nerve development.

## Materials and Methods

### Ethics Statement

This study was carried out in accordance with recommendations in the Guide for the Care and Use of Laboratory Animals of the National Institutes of Health. The protocol (2013–0115) was approved by the Institutional Animal Care and Use Committee of The Stowers Institute for Medical Research. Adult mice were euthanized via CO2 and cervical dislocation according to the recommendations of the American Veterinary Medical Association and all efforts were made to minimize any potential suffering.

### Mouse Lines

*Ptch1* ^*Wig*^, *Ptch1* ^*LacZ*^, *Wnt1Cre*, *R26RYFP*, *TOPgal* and *Hhat* ^*Creface*^ mice were maintained as described previously [8–14]. The morning of vaginal plug identification was defined as E0.5 for embryo collection and staging. We designated *Ptch1* ^*Wig/Wig*^ as *Wig* homozygous mutants and *Hhat* ^*Creface/Creface*^;*Ptch1* ^*Wig/Wig*^ as double-homozygous mice. Either wild-type or heterozygous littermates were used as control mice described in this study. Unless otherwise indicated, we used a minimum of 4 or 5 embryos from multiple distinct litters for each parameter analyzed in this study.

### Generation and identification of the *Ptch1* ^*Wig*^ mouse mutation

The *Wiggable (or Wig)* mutation was generated in a previously described N-ethyl-N-nitrosourea (ENU) screen for recessive mutations leading to craniofacial and neural tube defects [8]. Briefly, mutagenized fathers from a hybrid C57Bl/6/SV129J background were outcrossed to FVB females to generate founders. The male founders were subsequently mated to FVB females and the resulting daughters were backcrossed to the founders to identify recessive mutant phenotypes. One phenotype encompassed a kinked and dysmorphogenic neural tube in the craniofacial region and was termed *Wiggable* to reflect the superficial resemblance to Baroque period English wigs. DNA from mutant and control littermate embryos was collected along with tail biopsies from founder males and subjected to microsatellite and single nucleotide polymorphism (SNP) mapping, which narrowed down the affected region to mouse chromosome 13 (qB2 to qB3). This region is gene dense but contained only a few genes known to regulate embryonic neural development, including *Ptch1*. We used a candidate approach to sequence the genomic (intron/exon) regions of the genes residing in the minimal interval. Sequencing primers were designed to cover the entire mouse *Ptch1* gene (available upon request), which was one of our prioritized gene candidates in the minimal affected region, and we discovered a T to A substitution in the 3’end of intron 15 which segregated with the mutant phenotype (S1 Fig.).

### Validation of the *Ptch1* ^*Wig*^ mutation by qPCR

RNA was extracted using the RNeasy kit (Qiagen, Valencia, CA) and quantified using a Bioanalyzer. Primer Express v3.0 was used to make the primers and probes covering the splice junction between exons 15 and 16 of the *Ptch1* gene. Amplicon length was 92 base pairs (bp) for the wild type product and 99bp for the *Wig* mutant product. The following primers were used to generate the amplicon: CAA GCA ACT TCC CCA AAT GTG and ATC CTC CCA GTT TCC CAG TCA. *Actin* endogenous control probe and primers were ordered as a pre-designed assay from Applied Biosystems (Mouse ACTB (actin, beta) Endogenous Control # 4352933E). For the mutation site, the following dye-coupled sequences were used: *Ptch1* wild type probe: 6FAM—TGG CTT CAA GGA CTT C—MGBNFQ; *Ptch1* ^*Wig*^ mutant probe: VIC—ACT GGC TTC AAG TTT CTA GGA—MGBNFQ. The linear dynamic range of the qPCR assay was determined by making serial dilutions of the RNA and performing the reverse transcription (RT) reaction and subsequent amplification using the wild type, mutant and *Actin* endogenous control probes and primers. For the reverse transcription (RT) reaction, we utilized the high capacity cDNA reverse transcription kit from Applied Biosystems (Life Technologies, Grand Island, NY), using 100ng of RNA per reaction in a total volume of 20μL. Each sample was then split into 3 reactions where the wild type, mutant and endogenous control primers and probes were added. The cycling conditions for the cDNA amplification were: denaturation at 95°C for 2 min, followed by 40 cycles of 95°C for 15 seconds (sec) and 60°C for 1 minute (min). Each sample was genotyped by amplification of genomic DNA with forward and reverse primers detailed above using the following cycling conditions: 95°C for 2 min then 32 cycles of 95°C/30sec, 60°C/30 sec, and 72°C/30 sec.) The resulting amplicon was sequenced from both directions using the same primers and aligned to the expected sequence to identify the mutation polymorphism (S2 Fig.).

### Cloning of the *Ptch1* ^*Wig*^ mutation and Western blot

The sequencing of *Wiggable (Wig)* founders and their offspring confirmed the mutation introduced a 7 base pair insertion into the *Ptch1* cDNA (S1 Fig.). This in turn was predicted to generate a TGA codon 17 base pairs downstream of Exon 16, producing a premature truncation at position 848 in the mouse PTCH1 protein. To model the *Wig* mutation in cell culture, we used the QuikChange In Vitro mutagenesis kit (Agilent, Santa Clara, CA) to introduce the 7 bp insertion into the full-length mouse *Ptch1* cDNA which was cloned in the expression vector pcDNA3.1 (gift from Dr. Kazushi Aoto). The insertion was confirmed by sequencing. Wild type *Ptch1* or *Ptch1* ^*Wig*^ was transfected into Hek293T cell lines and after 48 hours culture, protein was extracted using a standard lysis buffer (20nM Tris HCl pH8, 137mM NaCl, 10% glycerol, 1% nonidet P-40, 2mM EDTA, protease inhibitors). Protein was quantified using a BSA curve and 40–60ng of protein was loaded per lane. Blots were transferred onto PVDF membranes (Bio-Rad, Hercules, CA) and blocked with 5% BSA solution. To detect PTCH1 protein, blots were probed with an amino-terminal specific PTCH1 antibody (G-19, Santa Cruz Biotechnology, Santa Cruz, CA; 1:1000).

### Complementation of the *Ptch1* ^*Wig*^ mutation with the *Ptch1* ^*LacZ*^ null allele

To test whether the *Wig* mutation was indeed a loss-of-function mutation in *Ptch1*, we conducted a functional complementation assay with *Ptch1* ^*LacZ/ +*^ mice (Jackson Laboratory strain 003081). The *Ptch1* ^*LacZ*^ allele contains a *lacZ-neo* fusion gene that disrupts endogenous *Ptch1* gene function [9]. *Ptch1* ^*LacZ/ LacZ*^ embryos exhibit neural tube abnormalities and are typically lethal at E9.5. *Ptch1* ^*Wig/+*^ heterozygotes were interbred with *Ptch* ^*LacZ/+*^ heterozygotes to generate embryos which were genotyped for the presence of both *Wig* and *LacZ* alleles. β-galactosidase staining (described below) was evaluated to determine the spatiotemporal expression of the *Ptch1* locus in the *Ptch1* ^*Wig/LacZ*^ mutants.

### Immunostaining and cell death detection

Whole-mount immunostaining of mouse embryos was performed as previously described [8,15] using antibodies against Neuronal Class III β-tubulin (TUJ1) (Covance, 1;1000), Neurofilament (Developmental Studies Hybridoma Bank, 2H3, 1:1000) and GFP (Invitrogen, 1:500). For section immunohistochemistry, embryos were fixed in 1% Formaldehyde in PBT overnight at 4°C and were processed through a sucrose gradient, embedded in Tissue-Tek (OCT compound, Sakura), and cut into 10-μm-thick sections. The slides were washed in TBST (TBS with 0.1% Tween) and blocked with 1% BSA in TBST for 1 hour at room temperature. Slides were incubated with anti-SOX10 (R&D systems, 1:50), anti-PAX3 (Developmental Studies Hybridoma Bank, 1:100), anti-TUJ1 (Covance, 1:1000), anti-GFP (Invitrogen, 1:500) with 1% BSA in TBST for overnight at 4°C. After several washes in TBST, the slides were incubated using the appropriate secondary antibody for 1 hour at room temperature and counterstained with DAPI (Sigma-Aldrich) (1:1,000) for 10 minutes at room temperature and mounted with fluorescent mounting medium (DakoCytomation). Apoptosis was visualized using the In Situ Cell Death Detection Kit Fluorescein (Roche) according to the manufacturer’s instructions.

### In situ hybridization

Whole-mount *in situ* hybridization and sectioning was performed as previously described [10].

### β-galactosidase staining

*TOPgal* reporter mice [11] were bred into the *Ptch1* ^*Wig/+*^ and *Hhat* ^*Creface/+*^;*Ptch1* ^*Wig/+*^ mice and embryos were stained for *LacZ* activity using the β-galactosidase Staining Solution Kit (Chemicon/Millipore) according to the manufacturer’s instructions.

## Results

### Truncated PTCH1 protein leads to excessive Shh signaling during mouse embryonic development

**Fig 1:**
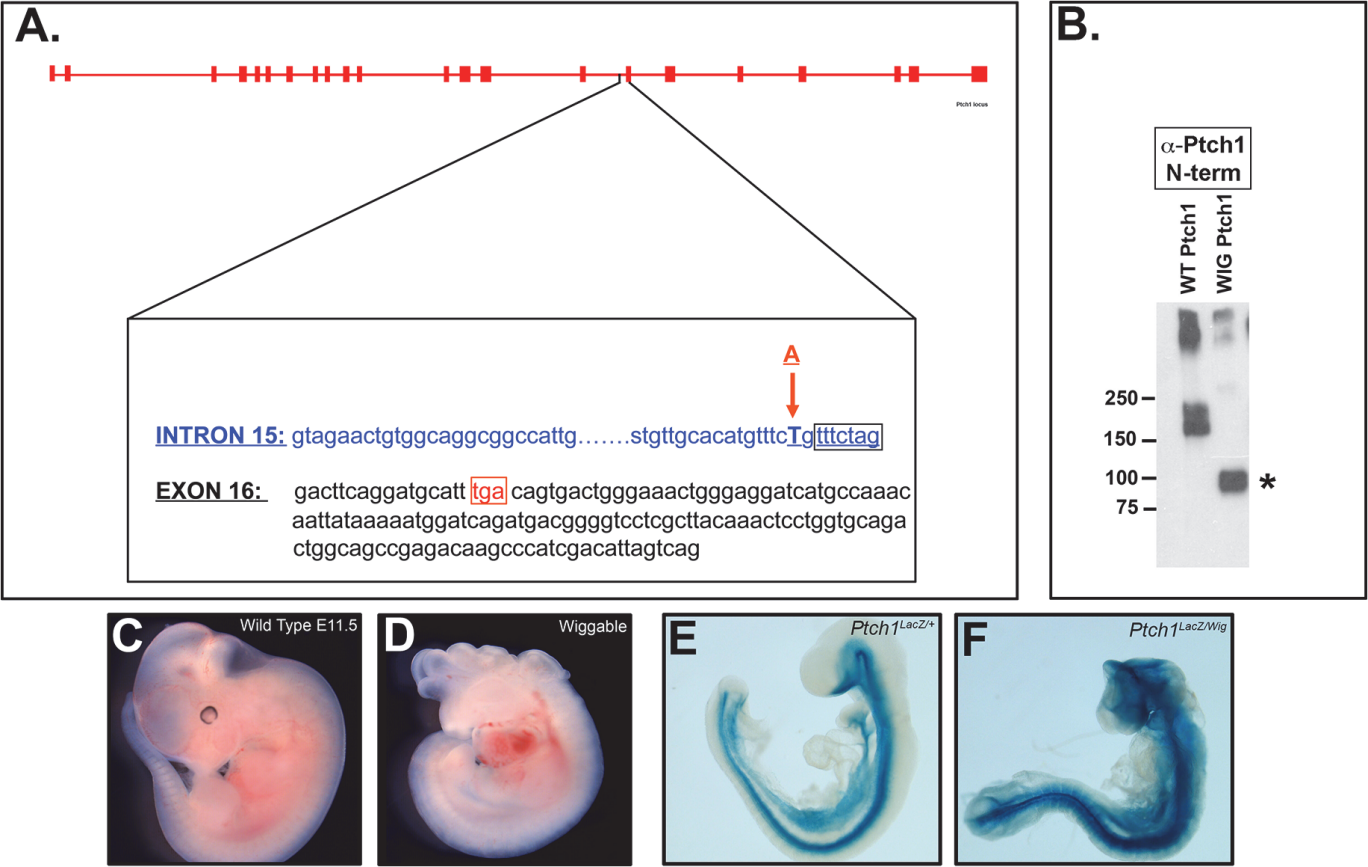
Generation of a novel Patched1 mutation by ENU mutagenesis. (A) Schematic depicting the *Patched1* (*Ptch1*) genomic structure indicating an A to T substitution in the 3’ region of intron 15, creating a new consensus splice acceptor site. The mutation is predicted to creates a 7 base pair insertion (box) in the 5’ end of Exon 16 that leads to a premature stop (TGA, box) 17 downstream. The mutation is referred to as *Wiggable* (or *Ptch1* ^*Wig*^). (B) Western blot of Hek293T cells overexpressing either wild type PTCH1 or *Wig* PTCH1 and probed with a PTCH1 amino (N)-terminal specific antibody. Wild type PTCH1 migrates as a complex centered at 170kDa. The *Wig* PTCH1 migrates at approximately 90kDa. (C-D) Whole mount images of wild type (C) and *Ptch1* ^*Wig/Wig*^ (D) embryos at E11.5, which display defects in craniofacial and neural development. (E) *LacZ* staining in a *Ptch1* ^*LacZ/+*^ heterozygote control showing *Ptch1* gene activity in ventral neural tissues and in endoderm. *Ptch1* ^*LacZ/+*^ display normal embryonic development (F) Complementation of the *Ptch1* ^*Lac*Z^ allele with the *Ptch1* ^*Wig*^ (*Ptch1* ^*LacZ/Wig*^) allele, leading to open neural tube and craniofacial defects and an upregulation of *Ptch1* ^*LacZ*^ gene activity.

Through an ENU mutagenesis screen for recessive embryonic phenotypes, we isolated a mutant we named *Wiggable (Wig)* due to a kinked and overproliferative neural tube that superficially resembled an English wig (Fig. 1) [8]. Microsatellite and SNP mapping narrowed the candidate region to mouse Chromosome 13 (qB2-qB3). We then focused on candidate genes within the interval known to have embryonic patterning defects. Among them was *Patched1* (*Ptch1)*, which encodes a receptor for Hedgehog ligands [9]. *Wig* ENU mutants segregated with a T to A substitution in the 3’end of intron 15 of *Ptch1*, which creates a novel splice acceptor site and produces a 7 base pair insertion in the 5’ end of Exon 16 (Fig. 1A, S1 Fig.). RT-PCR sequencing of cDNAs derived from *Wig* carrier mice embryos confirmed the presence of a 7 base pair insertion in the cDNA of *Ptch1* (S1 Fig.). This in turn created a premature stop codon17 base pairs into exon 16 which is predicted to truncate the mouse PTCH1 protein at amino acid position 848. The full-length mouse PTCH1 protein is 1424 amino acids, and the mutation truncates the protein within the 6th extracellular loop, keeping the sterol-sensing domain intact, but abrogating part of the Hedgehog-interacting extracellular domain. Since most of the C-terminal domain is lacking, the *Wig* ENU mutation is expected to create a *Ptch1* loss-of-function allele and abrogate PTCH1 inhibition of Smoothened signaling. In support of this idea, qPCR analysis of *Ptch1* expression revealed an approximately 4-fold increase in *Ptch1* locus transcription in *Ptch1* ^*Wig/Wig*^ mutants relative to wild-type controls (S2 Fig.). This reflects the fact that the *Ptch1* locus is under the regulatory control of GLI proteins, and also that *Ptch1* loss of function mutations result in the hyper-activation of Shh target genes [16].

The *Wig* allele of *Ptch1* was cloned to test whether it would create a truncated protein in transfected Hek293T cells. Using amino (N)-terminal-specific *Ptch1* antibodies to probe the resulting Western blot, we confirmed that the *Wig* allele of *Ptch1* did indeed produce a stable truncated form of PTCH1 that migrated at ∼90kDa (Fig. 1B). To further evaluate the effect of the *Wig* mutation on *Ptch1* mRNAs levels, we performed a qPCR experiment using mRNA extracted from E10.5 embryos obtained from *Ptch1* ^*Wig/+*^ intercrosses (S2 Fig.). *Ptch1* ^*Wig/Wig*^ mutants (red bars, specimens 216, 218, 219, and 224) expressed only the mutant form of *Ptch1*, with undetectable levels of wild type *Ptch1* mRNA expression (S2 Fig.). *Ptch1* ^*Wig/+*^ heterozygotes (Specimens 220–222) showed approximately equal levels of both the wild type and *Wig* mutant *Ptch1* mRNA. No *Ptch1* ^*Wig*^ mutant mRNA was detected in wild type embryos. Interestingly, in the *Ptch1* ^*Wig/Wig*^ embryos *Ptch1* mRNA levels were highly elevated, consistent with deregulated *Shh* signaling. This is because the *Ptch1* locus is under the direct transcriptional regulation of *Shh* signaling through the binding of GLI transcription factors to its promoter [16].

We next performed a genetic complementation assay of the *Ptch1* ^*Wig*^ allele with the *Ptch1* ^*LacZ*^ mutation [9]. The *Ptch1* ^*LacZ*^ mutation creates a null allele that in *Ptch1* ^*LacZ/LacZ*^ embryos leads to severe patterning defects and lethality at E9.5. In contrast the *Ptch1* ^*Wig*^ mutation is less severe, with *Ptch1* ^*Wig/Wig*^ embryos surviving until E11.5–12.5 (Fig. 1C,D), although this could be due in part to strain background differences. The *Ptch1* ^*LacZ*^ allele can also be used to monitor *Ptch1* locus activity via β-galactosidase staining, and is strongly expressed in ventral neural tissues and the endoderm in E9.5 control embryos (Fig. 1E). While *Ptch1* ^*LacZ/+*^ and *Ptch* ^*Wig/+*^ heterozygotes are normal (Fig. 1E), *Ptch1* ^*LacZ/Wig*^ mutants exhibited severe neural tube closure and brain patterning defects together with early embryonic lethality (Fig. 1F). The failure to complement confirmed that the *Wig* mutation is a novel loss-of-function allele of *Ptch1*. The fact that β-galactosidase staining was more intense in the *Ptch1* ^*LacZ/Wig*^ E9.5 embryos than in controls is indicative of elevated Hh signaling and is consistent with the *Ptch1* locus being a direct target of activated *Shh* signaling (Fig. 1F, S2 Fig.).

### *Ptch1* ^*Wig/Wig*^ embryos exhibit disrupted cranial nerve development

**Fig 2:**
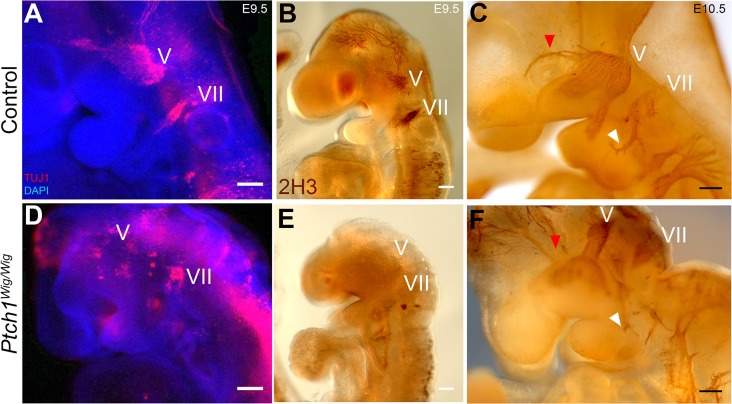
Defect of trigeminal (V) and facial nerve (VII) formation in *Ptch1* ^*Wig*^ mutant embryos. (A and D) Whole mount immunohistochemistry using anti-Neuronal Class III β-tubulin (TUJ1) in order to mark neuronal cells of cranial nerves (red) counterstained by DAPI (blue) of *Ptch1* ^*Wig*^ mutant vs. control E9.5 embryos. (B,C,E and F) Whole mount neurofilament (2H3) staining of E9.5 and E10.5 embryos. (C) The ophthalmic branch of the trigeminal nerve (red arrowhead), and the facial nerve (white arrowhead) were well developed in E10.5 wild type embryos. (F) *Ptch1* ^*Wig*^ mutants exhibited malformations of those nerves. Scale bars: 100μm (A,B,D and E); 200μm (C and F).

The survival of *Ptch1* ^*Wig/Wig*^ mutants until E11.5–12.5 afforded us the opportunity to examine the role of elevated *Shh* signaling in the formation and patterning of the cranial nerves. Immunohistochemistry on E9.5 control embryos revealed that the trigeminal (V) and facial nerves (VII) develop as clusters of Neuronal Class III β-tubulin (TUJ1) labeled neurons (Fig. 2A). In contrast, *Ptch1* ^*Wig/Wig*^ mutant embryos exhibited severely disorganized trigeminal and facial nerves (Fig. 2D). A similar phenotype could be seen via neurofilament (2H3) staining (Fig. 2B and E). By E10.5 the axonal branches of the trigeminal and facial nerves should have begun projecting to the distal facial tissues they eventually innervate as can be seen in control embryos (Fig. 2C). However, in *Ptch1* ^*Wig/Wig*^ mutant embryos the cranial nerves do not develop properly or project to their appropriate target tissues (Fig. 2F). These results suggest that the *Ptch1* ^*Wig/Wig*^ mutation perturbs cranial nerve development presumably in association with elevated *Shh* signaling.

### *Ptch1* ^*Wig/Wig*^ mutation results in cranial neural crest cell death

**Fig 3:**
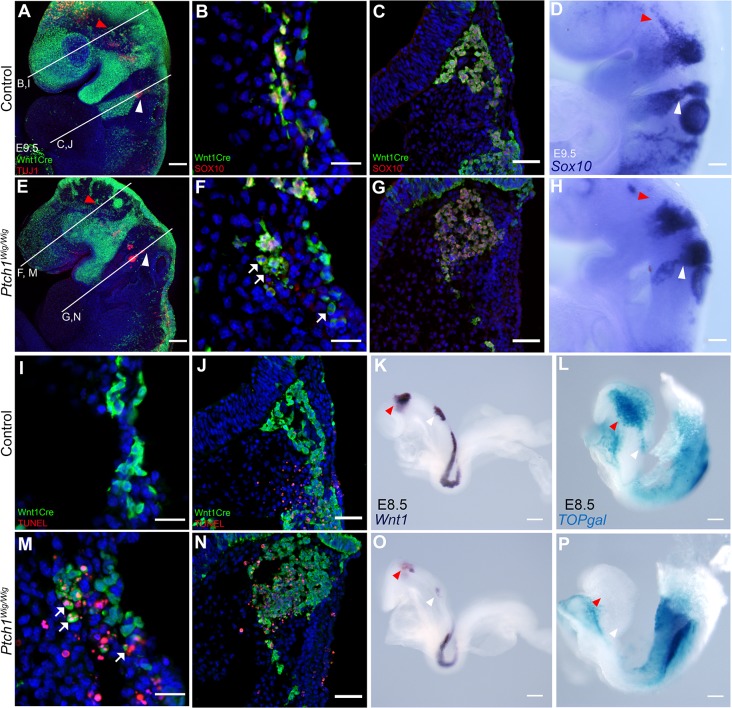
*Ptch1* ^*Wig/Wig*^ embryos exhibited increased cell death in cranial neural crest cells with down-regulation of survival factors. (A and E) Whole mount immunostaining of GFP (green) and TUJ1 (red) of *Ptch1* ^*Wig/Wig*^ vs. controls crossed with *Wnt1Cre;R26RYFP* reporter mice at E9.5. GFP-positive neural crest cells in the opV region in both control (A; red arrowhead) and *Ptch1* ^*Wig*^ embryos (E; red arrowhead). Fewer neural crest cells were detected entering the second branchial arch in *Ptch1* ^*Wig*^ embryos (E; white arrowhead) vs. controls (A; white arrowhead). (B,F,I and M) Horizontal section of opV and (C,G,J and N) facial nerve regions indicated in (A) and (E) stained for TUNEL (red; I,J,M and N) and SOX10 (red; B,C,F and G) GFP and DAPI (blue). SOX10 immunohistochemistry in opV region and facial nerve region of control (B and C) and *Ptch1* ^*Wig/Wig*^ embryos (F and G). (D and H) Whole mount *in situ* hybridization of *Sox10* of each genotype at E9.5. Elevated apoptosis in neural crest cells detected both in the opV region (I and M; white arrow) and facial nerve region (J and N) of *Ptch1* ^*Wig/Wig*^ mutants. (K and O) Whole mount *in situ* hybridization of *Wnt1* of *Ptch1* ^*Wig/Wig*^ (O) and control (K) at E8.5. (L and P) *TOPgal* activity (blue) of *Ptch1* ^*Wig/Wig*^ (P) and control (L) embryos at E8.5. Scale bars: 100μm (A,D,E and H); 20μm (B,F,I and M); 50μm (C,G,J and N); 200μm (K,L,O and P).

Cranial nerves are derived from both cranial neural crest cells and ectodermal placodes. The defects in cranial nerve development in *Ptch1* ^*Wig/Wig*^ mutant embryos could therefore reflect abnormal neural crest formation and migration, and/or aberrant placode cell development. To address the role of the cranial neural crest in the pathogenesis of the *Wig* phenotype, we crossed *Ptch1* ^*Wig/+*^ mice into *Wnt1Cre;R26RYFP* to genetically label cranial neural crest cells and their derivatives [13,14]. We did not observe any major defects in the neural crest cells emerging from the anterior hindbrain and migrating into the first pharyngeal arch in *Ptch1* ^*Wig/Wig*^ embryos (Fig. 3A vs Fig. 3E; red arrowhead). However, there were clearly far fewer migrating neural crest cells within the second pharyngeal arch and facial nerve regions in *Ptch1* ^*Wig/Wig*^ mutants compared to controls (Fig. 3A vs Fig. 3E; white arrowhead). We used Sox10 staining to further evaluate the effect of the *Wig* mutation on the migratory cranial neural crest cells. Interestingly, *Ptch1* ^*Wig/Wig*^ mutant embryos exhibited fewer SOX10 protein (Fig. 3B vs Fig. 3F; white arrow) and *Sox10 mRNA* (Fig. 3D vs. Fig. 3H; red arrowhead) positive neural crest cells specifically within the opV region relative to controls. However in contrast to the situation in the opV region, and taking into account the reduced number of GFP-positive neural crest cells in the facial nerve region, the relative proportion of SOX10/*Sox10* positive neural crest cells in *Ptch1* ^*Wig/Wig*^ mutants embryos was similar to controls (Fig. 3C vs Fig. 3G, Fig. 3D vs Fig. 3H; white arrowhead). Thus there is a consistent diminishment of neural crest cells in both the opV and facial nerve regions in *Ptch1* ^*Wig/Wig*^ compared to controls.

Ptch1 together with Hh signaling are collectively known to play critical roles in cell proliferation and survival [17–19]. Therefore we hypothesized that the diminished neural crest cell population in the opV and facial nerve regions of *Ptch1* ^*Wig/Wig*^ embryos may be due to apoptosis of neural crest cells. Indeed, TUNEL staining in combination with GFP labeling of neural crest cells revealed a substantial elevation of cell death specifically in neural crest cells in the opV region of *Ptch1* ^*Wig/Wig*^ mutants compared to control littermates (Fig. 3I vs Fig. 3M). A similar increase of cell death in GFP-positive neural crest cells was also observed in the facial nerve region of *Ptch1* ^*Wig/Wig*^ mutants compared to controls (Fig. 3J vs Fig. 3N). Consistent with these results, TUNEL staining together with SOX10 immunohistochemistry also revealed a significant elevation of cell death in cranial neural crest cells in *Ptch1* ^*Wig/Wig*^ mutants (S3 Fig.). In contrast, we did not observe any difference in apoptosis of TUJ1 positive neuronal cells either in the opV or facial nerve of control versus *Ptch1* ^*Wig/Wig*^ embryos (S3 Fig.). These findings demonstrate that aberrant cranial nerve development in *Ptch1* ^*Wig/Wig*^ embryos is associated with elevated apoptosis of migratory cranial neural crest cells but not of post-mitotic neuronal cells. Since the *Wig* mutation in *Ptch1* results in elevated Shh signaling, our results suggest that the spatiotemporal activity of this important mitogen has important consequences for the survival of migratory cranial neural crest cells during cranial nerve development.

We recently discovered that Shh signaling restricts the extent of canonical Wnt signaling in the developing craniofacial prominences [10]. Furthermore it has been well established that Wnt signaling is important for neural crest cell survival, as well as development and patterning of the cranial nerves [20–23]. Therefore we investigated the possibility that elevated Shh signaling in the *Ptch1* ^*Wig/Wig*^ mutants regulates canonical Wnt signaling in the migratory crest and/or placodes, leading to the pathogenesis of cranial nerve defects. Firstly, we performed *in situ* hybridization for *Wnt1*, which demarcates the dorsal neuroepithelial territory from which neural crest cells are derived and plays an important role in neural crest development and survival [23]. We found a marked down-regulation of *Wnt1* expression in the neuroepithelium of *Ptch1* ^*Wig/Wig*^ mutants (Fig. 3O vs Fig. 3K, arrowheads). Secondly, to further characterize the possibility of an association of perturbed canonical Wnt signaling with aberrant cranial nerve development in *Ptch1* ^*Wig/Wig*^ mutants, we crossed *Ptch1* ^*Wig/+*^ mice with the *TOPgal* reporter [11]. We observed a considerable reduction of *TOPgal* activity in *Ptch1* ^*Wig/Wig*^ mutant embryos compared to controls (Fig. 3P, vs. Fig. 3L, arrowheads). These observations are consistent with previous work that has shown that *Ctnnb1* (*β-catenin*) loss-of-function in migrating neural crest cells leads to neural crest cell death [22]. Collectively, these results illustrate that elevated Shh signaling restricts canonical Wnt signaling in cranial neural crest cells and influences their survival.

### *Ptch1* ^*Wig*^ mutants display defects in cranial placodal formation

**Fig 4:**
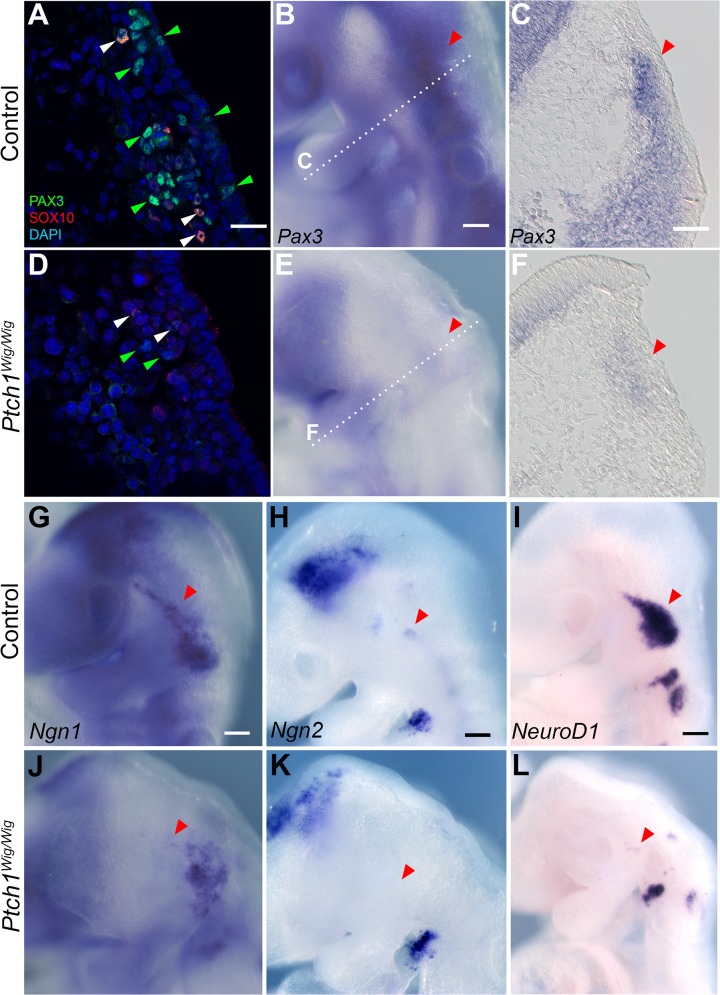
Enhanced Shh signaling in *Ptch1* ^*Wig/Wig*^ mutants affects cranial placode development. (A and D) Immunostaining of PAX3 (green), SOX10 (red), and DAPI (blue) in wild type (A) and *Ptch1* ^*Wig*^ mutants (D). PAX3-positive cells (placodal cells) and PAX3/SOX10 double-positive cells (neural crest cells) are identified by green and white arrowheads respectively. (B, C, E and F) *Pax3* mRNA expression in control (B and C) vs. *Ptch1* ^*Wig*^ mutants (E and F). Sections in (C and F) correspond to the dotted line in (B and E). Red arrowheads indicate reduced *Pax3* expression in *Ptch1* ^*Wig/Wig*^ mutant placodal primordia (E, F vs. B, C). (G-L) Whole mount *in situ* hybridization of indicated placodal markers *Ngn1* (G and J), *Ngn2* (H and K), and *NeuroD1* (I and L) on E9.5 wild type (G-I) vs. *Ptch1* ^*Wig*^ mutants (J-L). Red and white arrowheads identify the trigeminal and epibranchial placodes respectively. Scale bars: 20μm (A and D); 100μm (B,E and G-L); 50μm (C and F).

In addition to cranial neural crest cells, ectodermal placode cells provide major contributions to the development of the cranial ganglia [24]. We therefore explored whether cranial placode development was altered in association with elevated Shh signaling in *Ptch1* ^*Wig/Wig*^ mutant embryos. We used PAX3 and SOX10 co-immunostaining to define the placode cells of the trigeminal region [25], and *Ngn1*, *Ngn2*, and *NeuroD* expression via *in situ* hybridization as indicators of neuronal maturation in the placode [26]. We observed a marked diminution of PAX3-positive cells in *Ptch1* ^*Wig/Wig*^ embryos (Fig. 4A vs. Fig. 4D; green arrowhead). This reduction was further confirmed by *Pax3* mRNA *in situ* hybridization (Fig. 4B-C vs Fig. 4E-F). Similarly, *Ngn1*, *Ngn2* and *NeuroD* expression were all consistently down-regulated in the trigeminal placode and particularly in the opV branch in *Ptch1* ^*Wig/Wig*^ mutants compared to controls (Fig. 4G-I vs. Fig. 4J-L, red arrowhead). The downregulation of placodal markers in the *Ptch1* ^*Wig/Wig*^ mutants could be a result of aberrant development of placodal cells. This likely contributed to the defects in cranial nerve development in the mutants, together with the increased cell death in migrating cranial neural crest cells.

### *Ptch1* ^*Wig/Wig*^ embryos exhibit less integration between cranial neural crest cells and ectodermal placode cells

**Fig 5:**
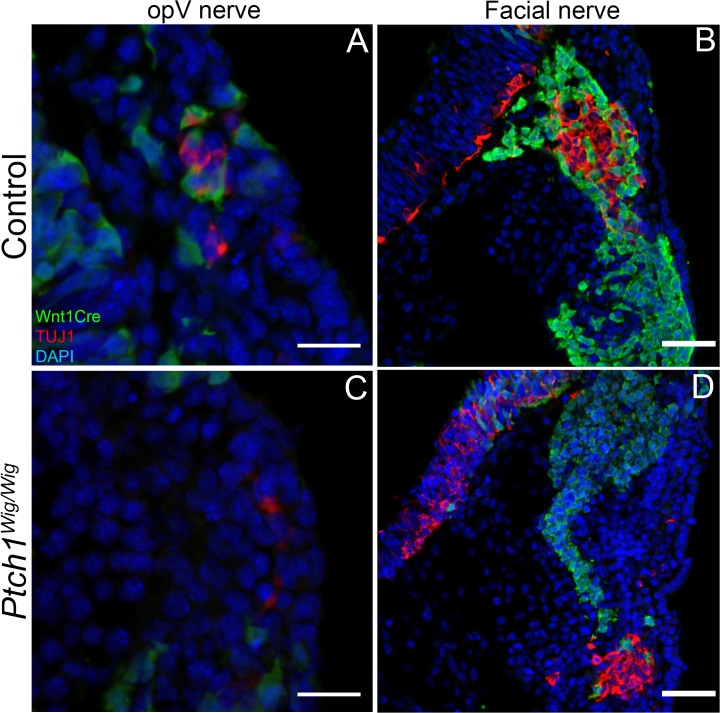
Increased Shh signaling in *Ptch1* ^*Wig*^ mutants resulted in less cellular interaction between neural crest and placodal cells. (A-D) *Wnt1Cre;R26RYFP* fate mapping in control wild type (A and B) and *Ptch1* ^*Wig/Wig*^ mutants (C and D) at E9.5. The YFP fate-mapped cells were identified by GFP immunostaining (green) and neurogenic placode cells were identified by TUJ1 staining (red) in the opthalamic (A and C) and facial nerve (B and D) region. The section planes in A-D are the same as those indicated in Fig. 3B,C,F and G. *Ptch1* ^*Wig/Wig*^ mutants (C and D) displayed much less neural crest cell (green) admixture within the ophthalmic and geniculate placodes relative to controls (A and B). Scale bars: 20μm (A and C); 50μm (B and D).

Cellular and molecular interactions between cranial neural crest cells and ectodermal placodes are essential for proper cranial nerve development [4]. Given that we observed defects in the survival of migrating cranial neural crest cells together with defects in placode development, we carefully examined the interactions between these two cell types during cranial nerve development via TUJ1 immunostaining combined with *Wnt1Cre;R26RYFP* in control and *Ptch1* ^*Wig/Wig*^ embryos. Clear cellular integration between neural crest cells and placode cells was observed in the developing trigeminal (Fig. 5A) and facial nerve of control E9.5 control embryos (Fig. 5B). In contrast, *Ptch1* ^*Wig/Wig*^ mutants exhibited considerably reduced mixing of migrating neural crest cells and placodal cells (Fig. 5C-D). Thus, increased *Shh* signaling in *Ptch1* ^*Wig/Wig*^ mutants, which results in the apoptosis of neural crest cells destined for the trigeminal and facial nerves is associated with the diminished integration between neural crest and placode cells. This contributes to the aberrant cranial nerve development observed in *Ptch1* ^*Wig/Wig*^ embryos compared to controls.

### Reduced *Shh* signaling restores canonical *Wnt* signaling and early cranial nerve development

**Fig 6:**
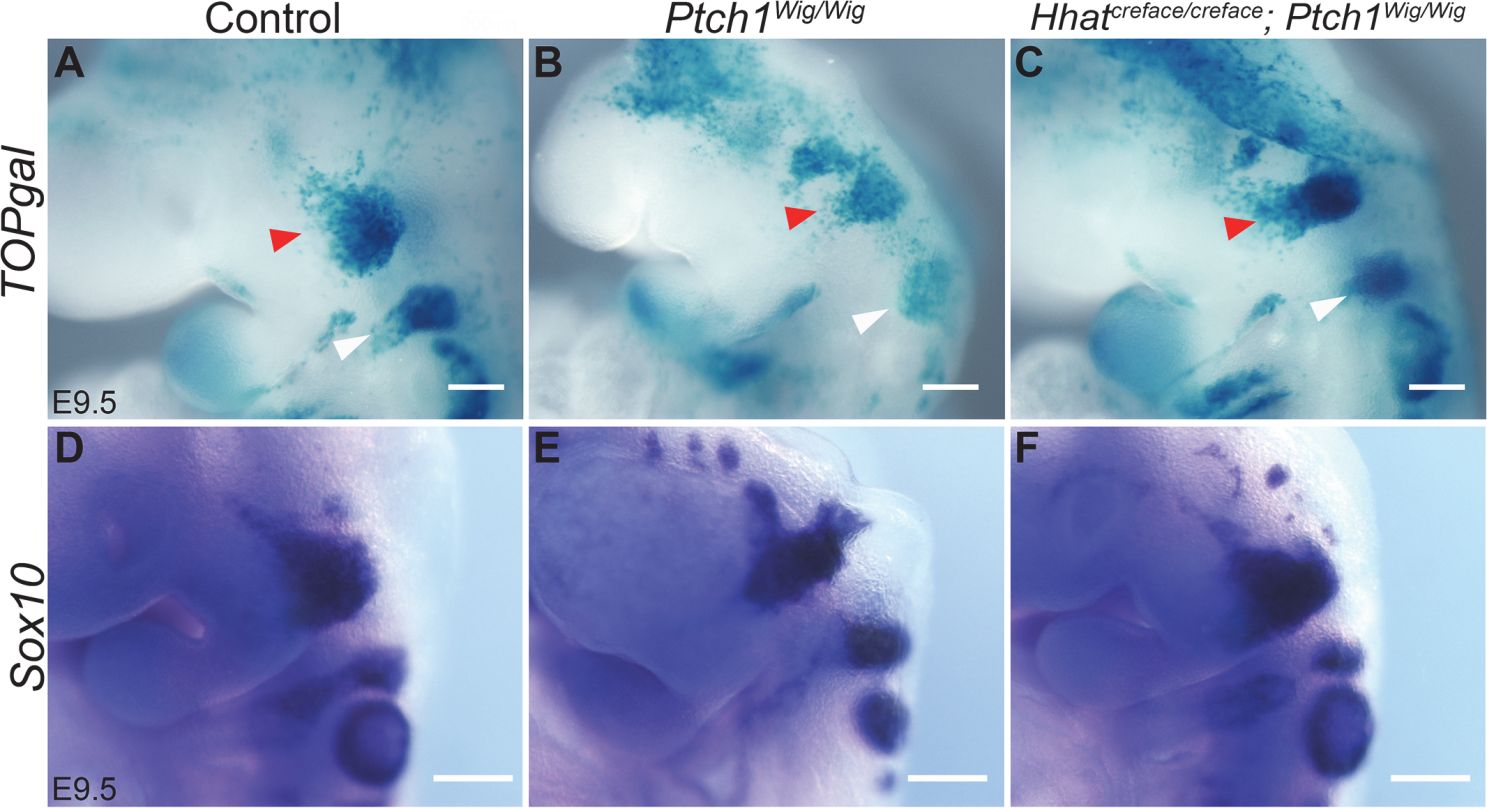
Restored *TOPgal* activity and cranial nerve development in *Hhat* ^*Creface/Creface*^; *Ptch1* ^*Wig/Wig*^ double mutants. (A-C) *TOPgal* expression in E9.5 controls (A), *Ptch1* ^*Wig/Wig*^ mutant (B), and *Hhat* ^*Creface/Creface*^;*Ptch1* ^*Wig/Wig*^ compound mutant (C) embryos. Red arrowheads indicate trigeminal nerve region and white arrowheads show the facial nerve region. (D-F) Whole mount *Sox10 in situ* hybridization in the indicated control or mutant embryos. (C and F) *Hhat* ^*Creface/Creface*^;*Ptch1* ^*Wig/Wig*^ double mutants showed a restoration of cranial nerve patterning indicated by *TOPgal* and *Sox10* staining levels similar to controls (A and D). Scale bars: 200μm.

The observation that elevated *Shh* signaling results in diminished canonical *Wnt* signaling in association with cranial nerve defects (Fig. 3K and L vs Fig. 3O and P) raised the idea that genetically reducing Shh signaling in *Ptch1* ^*Wig/Wig*^ embryos might restore Wnt signaling to normal and rescue cranial nerve development. We therefore used the *Hedgehog palmitoylase* (*Hhat*) loss-of function mutant (*Hhat* ^*Creface/Creface*^) to lower the levels of *Shh* signaling in *Ptch1* ^*Wig/Wig*^ mutants by producing compound mutants. The disruption of *Hhat* diminishes palmitoylation of SHH which perturbs its secretion and long range activity [12,27]. Furthermore, we also bred the *TOPgal* reporter into *Hhat* ^*Creface/Creface*^;*Ptch1* ^*Wig/Wig*^ compound mutant mice to visualize the spatiotemporal activity of canonical *Wnt* signaling. In E9.5 *Ptch1* ^*Wig/Wig*^ embryos, *Wnt* signaling as measured by *TOPgal* activity was diminished in the trigeminal (red arrowhead) and facial (white arrowhead) nerve regions (Fig. 6A-B). Strikingly, *TOPgal* activity was restored to wild-type levels in *Ptch1* ^*Wig/Wig*^;*Hhat* ^*creface/creface*^ compound mutant embryos (Fig. 6C). Furthermore, *Sox10 in situ* hybridization confirmed that while *Ptch1* ^*Wig/Wig*^ embryos exhibited reduced migrating neural crest cells and hypoplasia of the opV branch of the trigeminal nerve as well as the facial nerve (Fig. 6E), cranial nerve patterning was largely restored in *Ptch1* ^*Wig/Wig*^;*Hhat* ^*creface/creface*^ double mutants (Fig. 6F, compare with Fig. 6D). Collectively, these findings suggest that an appropriate balance between Shh and Wnt signaling is required to ensure the survival of cranial neural crest cells and proper development of the cranial neurogenic placodes which are essential for the orchestrated integration of these two cell populations during cranial nerve development.

## Discussion

Proper formation of the cranial nerves relies on dynamic tissue interactions mediated by multiple signaling pathways. In this study, we discovered that the interplay between neural crest cells and placodes cells depends on feedback regulation of Shh and Wnt signaling. We focused on patterning of the trigeminal and facial nerves because of their stereotypical morphology and uncovered a causal link between migratory neural crest cell survival and a balance between Shh signaling and the canonical Wnt pathway.

### Using *Ptch1* ^*Wig/Wig*^ mice to study the role of *Shh* signaling in early embryogenesis

*Ptch1* ^*LacZ*^ is a null allele of *Ptch1* that is commonly used to study the distribution, level and effects of perturbed Shh signaling during embryogenesis [9]. However, *Ptch* ^*LacZ/LacZ*^ homozygous mice are lethal at E9.5 and thus do not survive long enough to examine the effects of elevated Shh signaling on cranial nerve development. In contrast, *Ptch1* ^*Wig/Wig*^ homozygous mutants survive until around E12.5 (Fig. 1), which makes them amendable to study the effect of elevated Shh signaling on cranial nerve development.

### Elevated *Shh* signaling disrupts cranial nerve development via increased apoptosis of cranial neural crest cells

We observed that *Ptch1* ^*Wig/Wig*^ embryos exhibited disorganized trigeminal and facial nerve development (Fig. 2). These results are consistent with observations that ectopic SHH administration in chick embryos leads to abnormal trigeminal nerve development [28]. In order to a investigate the mechanisms underlying this phenotype, we first explored a role for apoptosis using TUNEL staining together with tissue specific markers and *Wnt1Cre;R26RYFP* reporter mice. Surprisingly, we detected no differences in cell death between control and *Ptch1* ^*Wig/Wig*^ embryos in the trigeminal or facial nerves (S3 Fig.). However, *Ptch1* ^*Wig/Wig*^ embryos exhibited substantially elevated levels of cranial neural crest cell apoptosis (Fig. 3 and S3 Fig.). These results are consistent with other observations that apoptosis of neural crest cells disrupts cranial nerve development in mice [29] and also that ablation of premigratory neural crest cells in chicken embryos results in disorganized trigeminal nerve formation [2,4,30,31]. Collectively, these findings argue that aberrant cranial nerve formation in *Ptch1* ^*Wig/Wig*^ mutant embryos arises primarily as a consequence of a diminished population of cranial neural crest cells, which are lost via apoptosis.

### Increased cell death in cranial neural crest cells in *Ptch1* ^*Wig*^ embryos associated with reduction of survival factors

Using the *Wnt1Cre;R26RYFP* reporter line [11,13,14] in combination with *Sox10* staining, we confirmed elevated apoptosis of cranial neural crest cells in *Ptch1* ^*Wig/Wig*^ embryos (Fig. 3). We noted the greatest reduction of SOX10 positive cells in *Ptch1* ^*Wig/Wig*^ embryos occurred in the opV region of the trigeminal nerve and was associated with the apoptotic loss of neural crest cells that typically invade that territory of the neurogenic placode. This prompted us to look for the source of survival signals that act downstream of Shh signaling to promote the survival of neural crest and placodal cells as they interact to form mature cranial nerves.

Elevated Shh signaling had been shown to restrict canonical Wnt signaling in various developmental contexts [10,32,33]. Moreover, Ctnnb1 is thought to function as a survival factor for migrating neural crest cells, and is necessary for activating canonical Wnt signaling in these cells [22,34]. Consistent with these observations, *Ptch1* ^*Wig/Wig*^ mutants display a reduction in canonical Wnt signaling in migrating cranial neural crest cells together with elevated cell death (Fig. 3). Furthermore, we succeeded in partially restoring cranial nerve development by reducing Shh signaling in *Hhat* ^*Creface/Creface*^;* Ptch1* ^*Wig/Wig*^ double mutants. In these double mutants, Hh ligands are not palmitoylated and secreted properly to form a long-range signaling gradient. In addition, because the *Wig* lesion results in a C-terminal truncation of Ptch1, Smoothened is presumably no longer inhibited and is therefore able to activate Shh signaling effectors. The compound *Hhat* ^*Creface/Creface*^;*Ptch1* ^*Wig/Wig*^ mutants exhibit a restoration of Shh signaling levels to that of normal embryos [10]. Consistent with this result, we observed a restoration of normal cranial nerve development in the *Hhat* ^*Creface/Creface*^;*Ptch1* ^*Wig/Wig*^ compound mutants, which was accompanied by a restoration of canonical Wnt signaling (Fig. 6). Collectively, these findings indicate that elevated Shh signaling in *Ptch1* ^*Wig/Wig*^ embryos leads to a down-regulation of canonical Wnt-dependent survival signaling, resulting in elevated neural crest cell death and consequently cranial nerve developmental defects.

### Possible pathway to inhibit trigeminal placode development from elevated *Shh* signaling

Ectodermal placodes are one of the two principal sources of cells that contribute to the cranial nerves with the other being the neural crest [1,2]. Pax3 is widely used as a marker of trigeminal placodal cells [25] and *Pax3* loss-of-function results in defects in trigeminal nerve development [35]. *Ptch1* ^*Wig/Wig*^ mutant embryos exhibited a significant reduction in *Pax3* expressing cells, consistent with a specific effect on trigeminal nerve development (Fig. 4). Interestingly, Wnt signaling [36,37] is required for *Pax3* expression in the trigeminal placode, and a recent study demonstrated direct regulation of *Pax3* by CTNNB1 [38]. Thus it is possible that the reduction of *Pax3* expression in *Ptch1* ^*Wig/Wig*^ embryos is due to the down-regulation of canonical Wnt signaling, which affects the regulatory events required for activation of the *Pax3* gene and subsequent placode development. It is important to note that other placodal markers such as *Ngn1*, *Ngn2* and *NeuroD1* [26] were also down-regulated in the trigeminal placode in *Ptch1* ^*Wig/Wig*^ embryos, but not in other placodes. This suggests that the development of individual cranial placodes may have different sensitivities to Shh signaling levels, with the trigeminal being particularly sensitive. It is also important to note altering Shh signaling affects the dorso-ventral patterning of neural tube [39], and this could potentially affect the patterning of neural crest cells emigrating out the neural tube boundary region as was observed in the case of *Zic5* loss-of-function [40]. This in turn can then have cascading effects on the proper development and patterning of the cranial nerves.

### Possible contribution of poor intermixed cranial neural crest and placodal cells to cranial nerve defects

**Fig 7:**
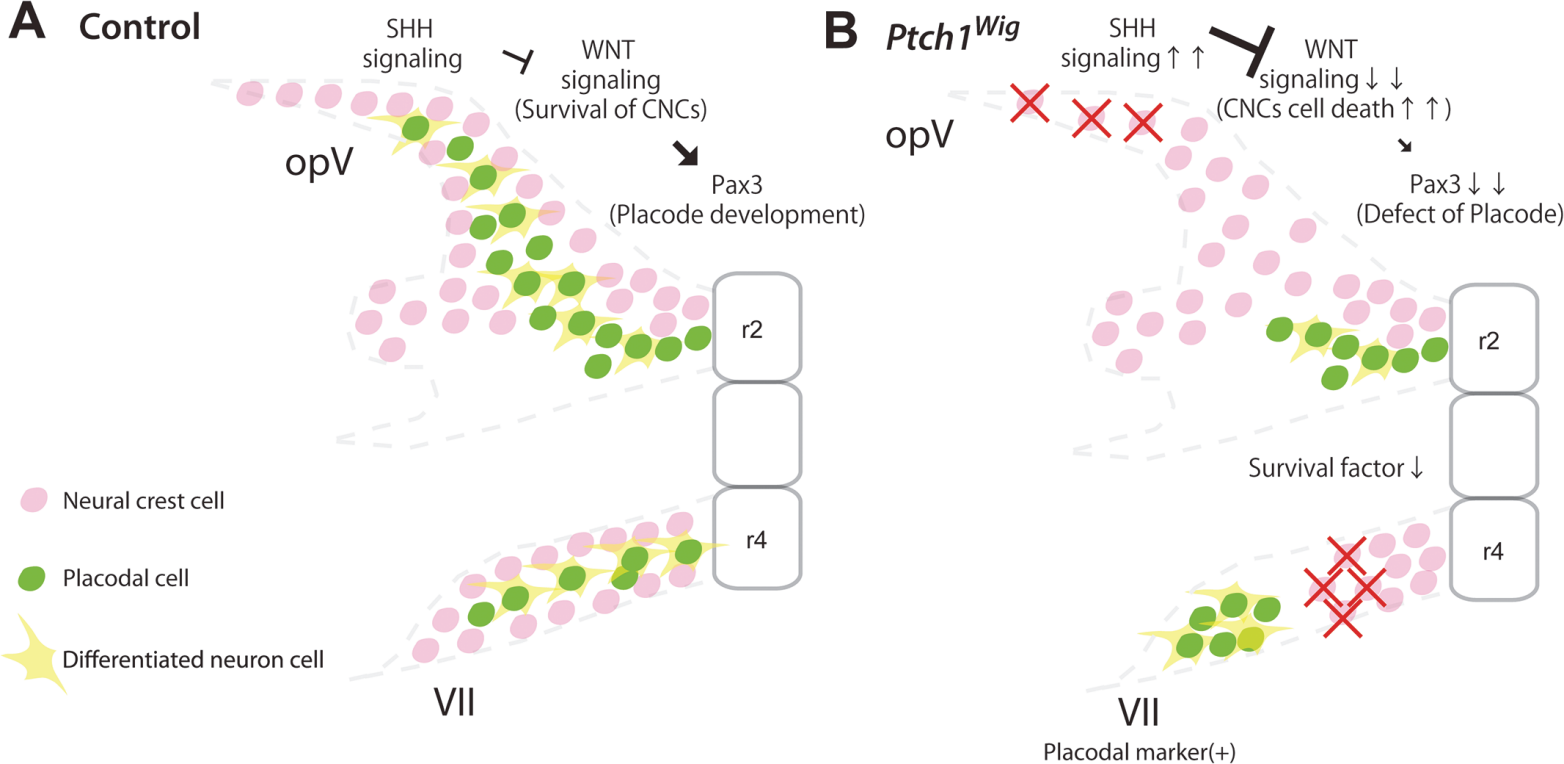
Schematic summary of elevated Shh signaling resulting in cranial nerve defects. (A) During normal development, neural crest cells migrating from rhombomere 2 (r2) or 4 (r4) interact with placodal cells to develop the cranial nerves. (B) *Ptch1* ^*Wig/Wig*^ embryos exhibit excessive *Shh* signaling leading to a reduction in neural crest cells and survival factors, which results in reduced neural crest cell-placode interactions and cranial nerve patterning defects.

In this study, we observed relatively poor integration between neural crest and neurogenic cells in the trigeminal and facial nerves of *Ptch1* ^*Wig/Wig*^ embryos compared to controls (Figs. 5 and 7). Recent studies have shown that neural crest cells play an important role during cranial nerve development by forming “corridors” or “conduits” which guide and surround neuronal cells [5,15]. Thus, any loss of migratory neural crest populations can impact cranial nerve formation by severing the cellular conduit for nascent axons growing out of the placodal cores of the cranial ganglia. In terms of molecular signals that can orchestrate cranial neural crest cell and placodal interactions, several studies have shown that cell adhesion molecules such as *N-cadherin* (*Cdh2*) or *αN-catenin* (*Ctnna2*) are important for cranial nerve integration [6,41]. Excessive *N-cadherin* (*Cdh2*) expression in *Xenopus* results in abnormal aggregation and migration of neural crest cells [42]. This suggests that inadequate cell adhesion in neural crest cells could affect the integration of neural crest and placodal cells. Furthermore, CTNNB1 can regulate cell adhesion by interacting with Cadherins [43]. Collectively, these studies suggest that the disorganized cranial nerves in *Ptch1* ^*Wig/Wig*^ embryos may also be due in part to disrupted Wnt signaling-dependent cell adhesion in cranial neural crest or/and placode cells, in addition to the reduced numbers of neural crest cells that is caused by apoptosis.

Elevated Shh signaling results in various well characterized craniofacial anomalies including anencephaly, hyperterolism, and cleft lip and palate [10,44,45] as well as a disease spectrum in humans called congenital cranial dysinnervation disorders, represented by Duane retraction syndrome (DRS) or Moebius syndrome (MBS) [46,47]. MBS is characterized by specific cranial nerve defects including trigeminal, abducens (VI) and facial nerves together with hyperterolism, which is a phenotype known to correlate with elevated Shh signaling [48,49]. Our results indicate that the co-ordinated integration of Shh and Wnt signaling plays a key role in promoting the survival and interaction of neural crest cells and ectodermal placodes during cranial nerve development. Moreover, perturbation of these molecular and cellular interactions may underpin the etiology of some congenital cranial dysinnervation disorders.

In conclusion, we have demonstrated a role for cross-talk between Shh and Wnt signaling in neural crest cells and placodes cells during cranial nerve development. Elevated Shh signaling did not affect the formation and migration of cranial neural crest cells, but did result in selective death of migratory neural crest cells. Elevated Shh signaling also perturbed the development of the neurogenic placodes, particularly the trigeminal placode. Together these events disrupted neural crest and placode cell integration and led to aberrant cranial nerve development. Shh signaling restricts canonical Wnt signaling, and reduced Wnt signaling is associated with neural crest cell death and abnormal cranial nerve development. Using genetic approaches to reduce the levels of elevated Shh signaling, we were able to restore the canonical Wnt signaling in the neural crest and placodal territories of the developing head and rescue cranial ganglia morphogenesis.

## Supporting Information

S1 Fig Schematic of the *Ptch1* ^Wig^ intronic mutation. The *Ptch1* ^*Wig*^ mutation creates a novel consensus splice acceptor site at the 3’ end of intron 15 due to A to T substitution. This in turn leads of a 7 base pair insertion (gtttctag) and premature truncation (tga) 17 base pairs downstream of the 5’ end of Exon 16 of the *Ptch1* gene. Sequencing of cDNAs derived from the biopsies of *Wig* carrier mice confirmed the presence of the predicted 7 base pair insertion.
(TIF)

S2 Fig Quantitative (q) PCR for *Ptch1* ^Wig^ and wild type *Ptch1* transcripts in E10.5 embryos. (A) Bar chart of the qPCR levels in *Ptch1* ^*Wig/Wig*^ mutants (red), *Ptch1* ^*Wig/+*^ heterozygotes or *Ptch1* ^*+/+*^ wild type (grey) E10.5 embryos. Embryos derived from *Ptch1* ^*Wig/+*^ intercrosses were lysed and subjected to RT-PCR. Primers specific for the *Wiggable* mutation site in exon 16 were used in qPCR experiments, with values normalized to *β-actin* transcript levels. Values were plotted as +/- standard deviation. *Ptch1* ^*Wig/Wig*^ mutants did not display significant levels of wild type *Ptch1* transcripts, and show an upregulation of *Ptch1* ^*Wig*^ levels. This upregulated *Ptch1* locus activity was only present in *Ptch1* ^*Wig*^ mutants and not in herteozygotes. (B) Raw data for the *Ptch1* wild type and *Wig* signal obtained from various embryo specimens.
(TIF)

S3 Fig Cell death of neuronal cells and neural crest cells in *Ptch1* ^Wig/Wig^ mutant embryos. (A and F) Whole mount immunostaining of Neuronal Class III β-tubulin (TUJ1) (red) and DAPI (blue). (B-E and G-J) Horizontal sections across the indicated planes in (A) and (F) of the ophthalmic (B,C,G and H) and facial nerve (D,E,I and J) immunostained for TUJ1 (red; B,G,D and I) or SOX10 (red; C,H,E and J), along with TUNEL (green) and DAPI (blue). No difference in cell death between control (B and D) and *Ptch1* ^*Wig*^ mutant embryos (G and I) in the ophthalmic and facial nerve. There was increased cell death in SOX10-positive migratory neural crest cells in *Ptch1* ^*Wig*^ mutants (H and J; white arrowhead) relative to controls (C and E). (K) No statistically significant difference in neuronal cell death numbers between control and *Ptch1* ^*Wig/Wig*^ embryos. (L) *Ptch1* ^*Wig/Wig*^ embryos showed significantly increased number of apoptotic SOX10-positive neural crest cells in the ophthalmic region relative to controls. Scale bars: 100μm (A and F); 20μm (B,C,G and H); 50μm (D,E,I and J). *P < 0.05, Student’s t test. Data are represented as mean ± SEM.
(TIF)
