# Supplementary figures and images for: Cranial Nerve Development Requires Co-Ordinated Shh and Canonical Wnt Signaling
Source: PLoS One. 2015 Mar 23;10(3):e0120821. doi: 10.1371/journal.pone.0120821 (PMC4370424; doi:10.1371/journal.pone.0120821)

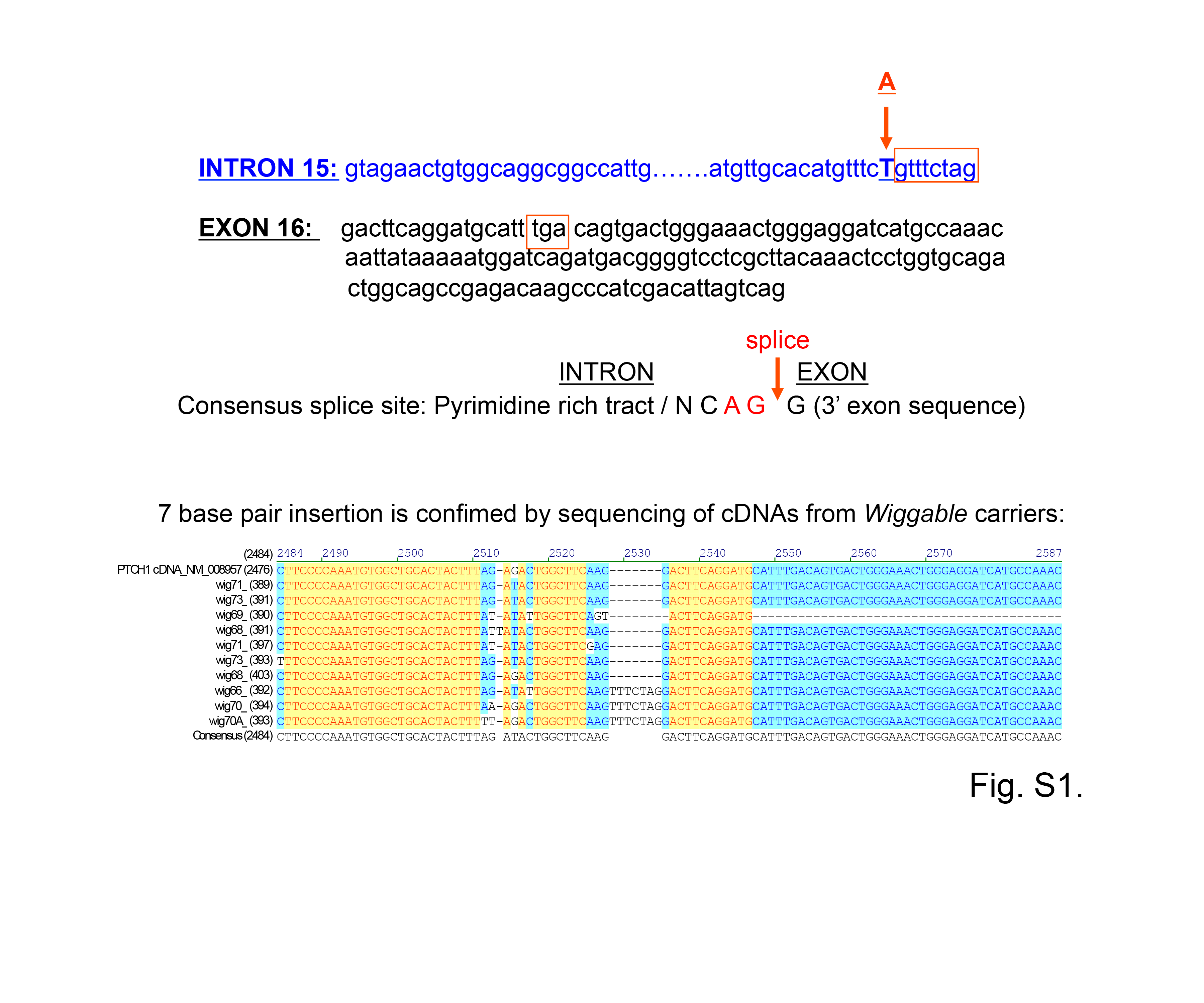

Supplement: S1 Fig — The Ptch1 Wig mutation creates a novel consensus splice acceptor site at the 3’ end of intron 15 due to A to T substitution. This in turn leads of a 7 base pair insertion (gtttctag) and premature truncation (tga) 17 base pairs downstream of the 5’ end of Exon 16 of the Ptch1 gene. Sequencing of cDNAs derived from the biopsies of Wig carrier mice confirmed the presence of the predicted 7 base pair insertion. (TIF) [file pone.0120821.s001.tif]

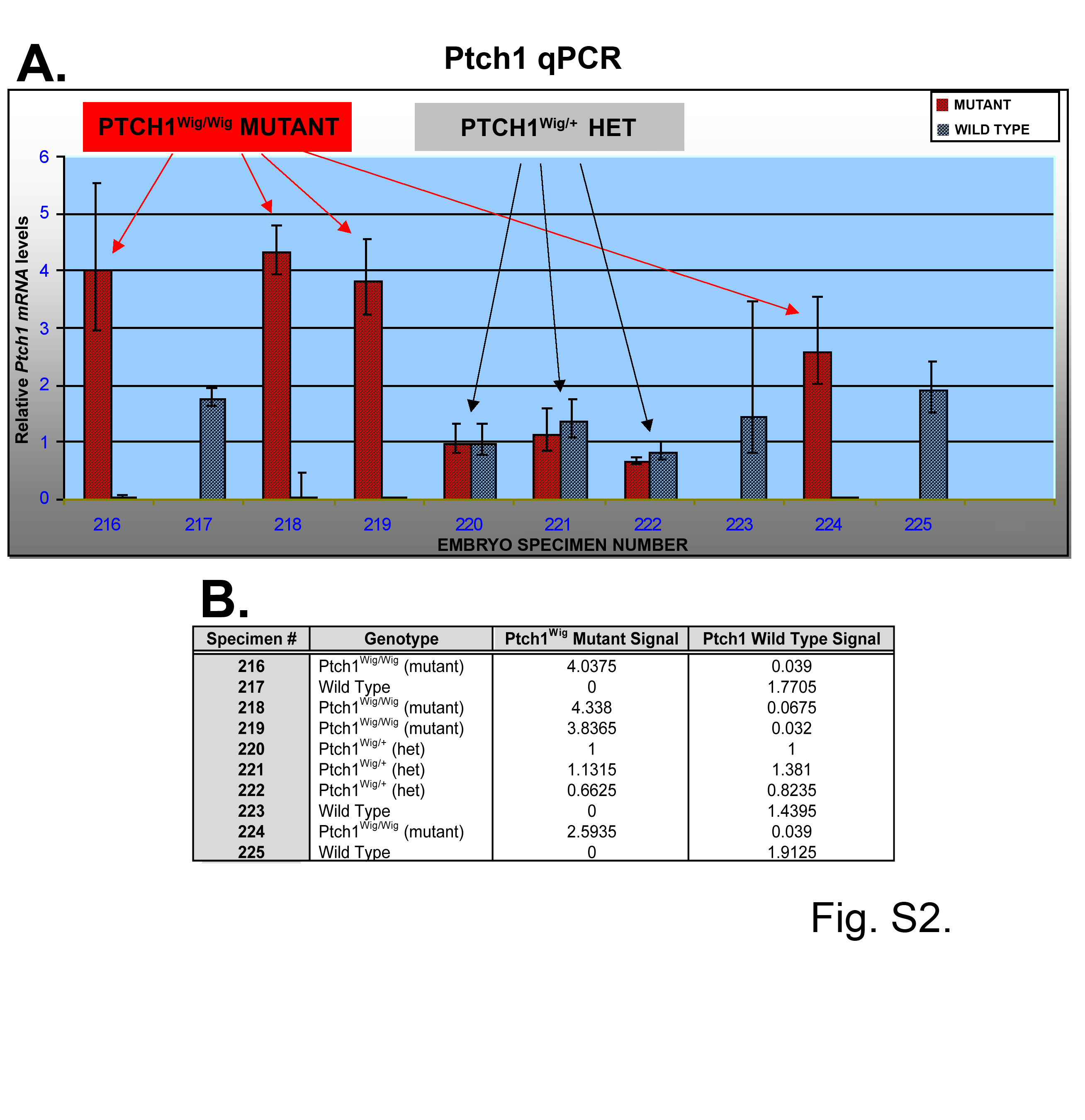

Supplement: S2 Fig — (A) Bar chart of the qPCR levels in Ptch1 Wig/Wig mutants (red), Ptch1 Wig/+ heterozygotes or Ptch1 +/+ wild type (grey) E10.5 embryos. Embryos derived from Ptch1 Wig/+ intercrosses were lysed and subjected to RT-PCR. Primers specific for the Wiggable mutation site in exon 16 were used in qPCR experiments, with values normalized to β-actin transcript levels. Values were plotted as +/- standard deviation. Ptch1 Wig/Wig mutants did not display significant levels of wild type Ptch1 transcripts, and show an upregulation of Ptch1 Wig levels. This upregulated Ptch1 locus activity was only present in Ptch1 Wig mutants and not in herteozygotes. (B) Raw data for the Ptch1 wild type and Wig signal obtained from various embryo specimens. (TIF) [file pone.0120821.s002.tif]

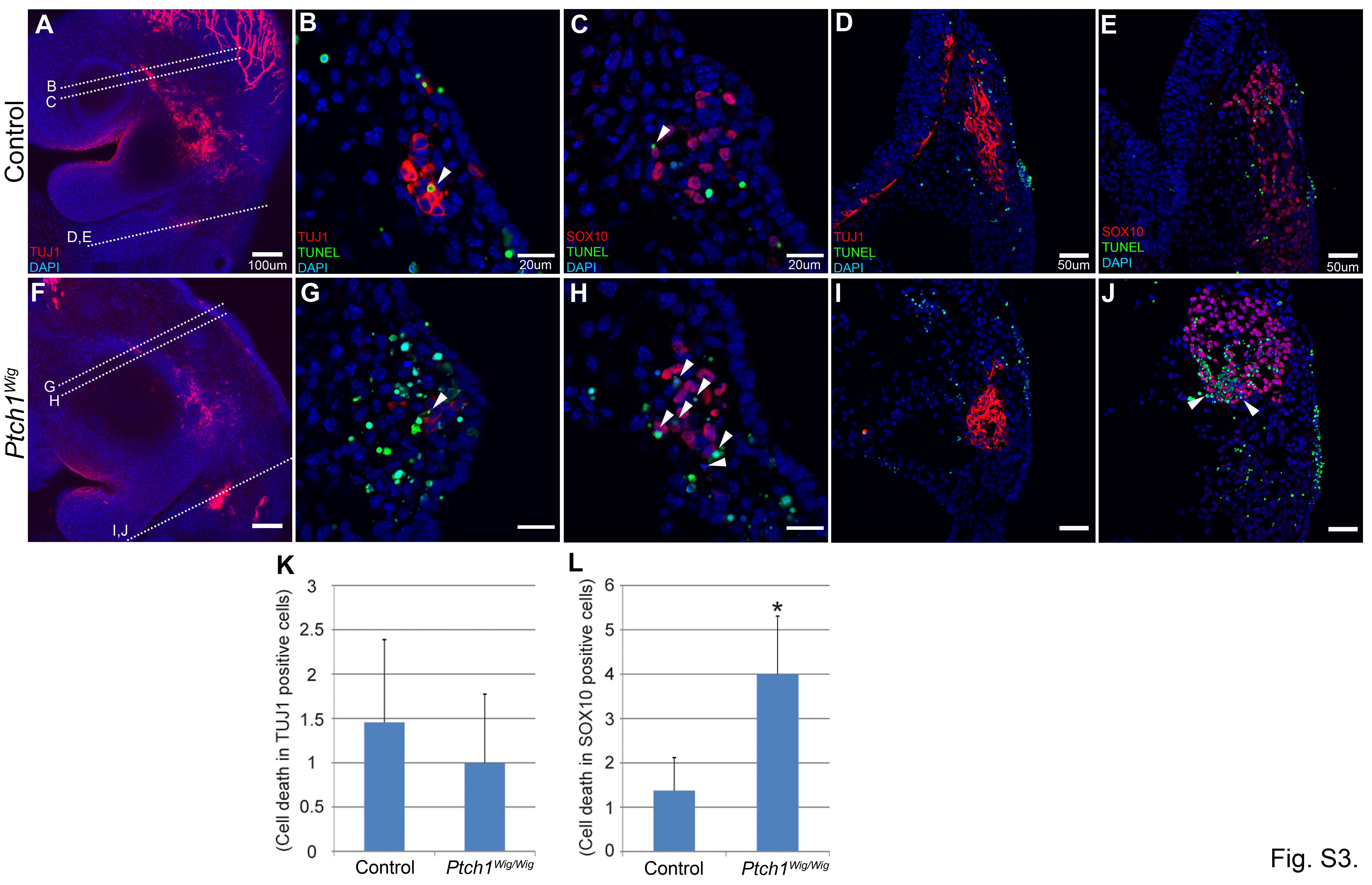

Supplement: S3 Fig — (A and F) Whole mount immunostaining of Neuronal Class III β-tubulin (TUJ1) (red) and DAPI (blue). (B-E and G-J) Horizontal sections across the indicated planes in (A) and (F) of the ophthalmic (B,C,G and H) and facial nerve (D,E,I and J) immunostained for TUJ1 (red; B,G,D and I) or SOX10 (red; C,H,E and J), along with TUNEL (green) and DAPI (blue). No difference in cell death between control (B and D) and Ptch1 Wig mutant embryos (G and I) in the ophthalmic and facial nerve. There was increased cell death in SOX10-positive migratory neural crest cells in Ptch1 Wig mutants (H and J; white arrowhead) relative to controls (C and E). (K) No statistically significant difference in neuronal cell death numbers between control and Ptch1 Wig/Wig embryos. (L) Ptch1 Wig/Wig embryos showed significantly increased number of apoptotic SOX10-positive neural crest cells in the ophthalmic region relative to controls. Scale bars: 100μm (A and F); 20μm (B,C,G and H); 50μm (D,E,I and J). *P < 0.05, Student’s t test. Data are represented as mean ± SEM. (TIF) [file pone.0120821.s003.tif]
